# The importance of service‐users’ perspectives: A systematic review of qualitative evidence reveals overlooked critical features of weight management programmes

**DOI:** 10.1111/hex.12657

**Published:** 2018-03-14

**Authors:** Katy Sutcliffe, G. J. Melendez‐Torres, Helen E. D. Burchett, Michelle Richardson, Rebecca Rees, James Thomas

**Affiliations:** ^1^ EPPI‐Centre UCL Institute of Education University College London London UK; ^2^ Division of Health Sciences Warwick Medical School University of Warwick Coventry UK; ^3^ Faculty of Public Health & Policy London School of Hygiene & Tropical Medicine London UK

**Keywords:** qualitative synthesis, service‐user perspectives, systematic review, weight management programmes

## Abstract

**Background:**

Extensive research effort shows that weight management programmes (WMPs) targeting both diet and exercise are broadly effective. However, the critical features of WMPs remain unclear.

**Objective:**

To develop a deeper understanding of WMPs critical features, we undertook a systematic review of qualitative evidence. We sought to understand from a service‐user perspective how programmes are experienced, and may be effective, on the ground.

**Search strategy:**

We identified qualitative studies from existing reviews and updated the searches of one review.

**Inclusion criteria:**

We included UK studies capturing the views of adult WMP users.

**Data extraction and synthesis:**

Thematic analysis was used inductively to code and synthesize the evidence.

**Main results:**

Service users were emphatic that supportive relationships, with service providers or WMP peers, are the most critical aspect of WMPs. Supportive relationships were described as providing an extrinsic motivator or “hook” which helped to overcome barriers such as scepticism about dietary advice or a lack confidence to engage in physical activity.

**Discussion and conclusions:**

The evidence revealed that service‐users’ understandings of the critical features of WMPs differ from the focus of health promotion guidance or descriptions of evaluated programmes which largely emphasize educational or goal setting aspects of WMPs. Existing programme guidance may not therefore fully address the needs of service users. The study illustrates that the perspectives of service users can reveal unanticipated intervention mechanisms or underemphasized critical features and underscores the value of a holistic understanding about “what happens” in complex psychosocial interventions such as WMPs.

## 1 INTRODUCTION

Obesity poses one of the greatest public health challenges for the 21st century.^1^ England, along with the rest of the UK, has one of the highest rates of obesity in the developed world.^2^ There has been extensive research effort in examining the impact of weight management programmes (WMPs) on people with obesity and those who are overweight, including a number of systematic reviews, for example^3^, ^4^, ^5^ and even reviews of reviews.^1^, ^6^ This high‐level evidence has established that programmes which address both diet and exercise are broadly effective for helping individuals to lose weight; that is, reviews consistently show pooled effects for weight loss that, despite high heterogeneity, suggest a meaningful and statistically significant impact for example.^5^ However, it remains unclear exactly what is important to shape effectiveness—or ineffectiveness—in these interventions. WMPs are often described in health promotion guidance or trial reports in relation to specific advice and education around both diet and exercise^7^ and a range of behavioural change techniques such as goal setting.^7^, ^8^ However, assessments of these educational and behavioural components have not produced clarity about which components of WMPs are instrumental in helping participants to lose weight. Detailed behavioural change taxonomies^9^ used in prior reviews to explore heterogeneity of intervention effect^5^ have been unable to explain the large amount of variation in weight loss between different programmes.

Developing a deeper understanding of the pathways to effectiveness, or ineffectiveness, may thus be important. Psychosocial interventions such as WMPs are particularly complex because intervention impacts will likely be affected by the nature and beliefs of both the provider and the recipient of the intervention.^10^ These ideas underlie recent calls for systematic reviews that ask not only “what works” but “what happens” when an intervention is implemented in a particular context, or with a particular population.^11^, ^12^, ^13^ Drawing on experiential evidence about “what happens”^14^, ^15^ enables us to unpick how an intervention is experienced and the influences of social interactions, context or the research process. Unpicking these components is an important, albeit challenging, task in understanding complex interventions and predicting what may work in the future, or in other contexts. Developing hypotheses from the ground up—that is, by listening to what service users and providers have to say—represents an opportunity to develop programme theories that best account for how programmes are experienced and are effective, on the ground.

Drawing on this logic, we undertook a mixed‐method systematic review, funded by the Department of Health England, to address the following question: What are the programme characteristics—and combinations of characteristics—that are associated with successful weight loss?^16^ This article focuses on the findings of the first stage of the review in which we drew on qualitative evidence to answer the question “What do WMP users and providers feel are the critical features of WMPs and how are these features perceived to impact on weight loss?” Whilst provider views largely underscored the views of service users, this article focuses specifically on the findings of the synthesis of service‐users’ views to illustrate how critical service‐user voices can be for understanding complex interventions. Whilst other qualitative evidence syntheses have examined broader user and provider views on, for example, obesity services in primary care^17^ and how WMPs are commissioned, run and viewed,^18^ our aim was more specific; we sought to focus in depth on the WMP features perceived to be critical for successful weight loss. In the second stage of the review, we examined evaluations of WMPs, employing qualitative comparative analysis (QCA) to test whether the features and mechanisms perceived to be important are actually associated with greater weight loss.^19^ The aim of this article was to reveal how experiential evidence of interventions, such as weight management interventions, may be vital to understanding their critical features. The findings of the overarching project, which are available online,^16^ further demonstrate the utility of this analytic approach.

## 2 METHODS

### 2.1 Study identification

Since a recent NICE (2013) review had undertaken searches for views studies,^18^ we assessed each of the qualitative studies included in that review. In addition, to identify qualitative studies conducted since that review was undertaken, we reran the search strategy employed for the NICE review on the highest yielding databases from their original search (MEDLINE, EMBASE, Web of Science, Medline in process) for the period from 2012 to 2014. To supplement the updated search, we also identified studies from other recent reviews of qualitative views research,^20^, ^21^, ^22^ conducted “citation chasing” on all studies meeting our inclusion criteria and contacted key authors in the field.

Studies returned by the search strategy were independently screened by pairs of reviewers (KS, RR, MR) using the predefined criteria specified in Table 1. All disagreements were resolved through discussion. Where full‐text papers were not easily retrievable (locally or from the British Library), authors were contacted.

**Table 1:** Criteria for inclusion of qualitative studies in the review

Criteria	Specification for inclusion and exclusions
Population	Inclusion: Adult (≥18 years) service users who had experience of attending a WMP^a^
Study type	Inclusion: Studies capturing the views, perceptions or beliefs about WMPs.
Country	Inclusion: UK
Language	Inclusion: English only
Quality	Exclusion: Conference abstracts; Study with a poor description of the methods; Studies with limited data on experience with WMP

a This paper focuses only on service users; however, the overarching review (16) also included service providers’ views.

### 2.2 Appraisal and in‐depth review

There is a lack of consensus among qualitative researchers about how to measure quality in qualitative research;^23^ therefore, we were cautious about excluding papers on the basis of quality. Nonetheless, to ensure a basic level of quality, papers were excluded if they (i) did not provide a clear account of the methods used for data collection and analysis; and (ii) contained only minimal or “thin” data pertinent to the review question. Thematic analysis^24^ was used inductively to code and describe the papers. The process involved reading and rereading the papers and applying line‐by‐line coding to capture descriptive themes about WMP features. These descriptive themes were then collapsed and developed to produce higher‐order analytic themes. Ten papers were initially coded and the themes scrutinized by the study team for conceptual coherence. Themes were collapsed where redundant or overlapping and split when necessary to improve their conceptual clarity. Definitions for each of the themes were written and were applied to all the studies to extract views data. New descriptive themes were added where they were not covered by the existing framework, which was modified on an iterative basis. To assess the relative importance of different WMP features, we examined (i) the number of studies commenting on them; (ii) the consistency of opinion regarding them; and (iii) how emphatically participants described their importance.

## 3 RESULTS

### 3.1 Included study characteristics

We identified 21 studies reported in 26 papers^25^, ^26^, ^27^, ^28^, ^29^, ^30^, ^31^, ^32^, ^33^, ^34^, ^35^, ^36^, ^37^, ^38^, ^39^, ^40^, ^41^, ^42^, ^43^, ^44^, ^45^, ^46^, ^47^, ^48^, ^49^, ^50^ that reported service‐user views on WMPs (see Figure S1 for PRISMA figure presenting flow of studies through the review). The 21 studies reported the views of 507 service users, and there was a good range in terms of gender, age and socio‐economic status, but limited data from minority‐ethnic service users; just one study focused on a programme specifically for a minority‐ethnic group^46^ and most others which provided information on the ethnicity of participants stated that they were predominantly white British. The vast majority of interviews were described as “semi‐structured”; these included individual face‐to‐face interviews, focus groups or telephone interviews. Most studies cited “thematic analysis” as their data‐analysis approach, 3 described using “content analysis,”^31^, ^42^, ^50^ 2 cited framework analysis,^26^, ^45^ and one described a “discourse analytic approach.”^33^ As noted above, this article does not report findings on service provider views, as these are reported elsewhere.^16^ Table 2 provides an overview of the participants and programmes for each included study. Most studies examined the views of service users on their experiences of a specific programme, but 4 studies asked participants to focus on a range of previous experiences.^30^, ^32^, ^33^, ^35^ Different service models were discussed including commercial or “for profit” services; community services, that is not‐for‐profit services based in community rather than health‐care settings; and health service‐based programmes, that is those services provided directly by National Health Service staff in a health service setting. The specific programmes discussed were largely delivered face‐to‐face, but 2 were delivered remotely via telephone or the Internet.^34^, ^35^ As Table 2 illustrates, roughly equal proportions of the studies focused on group‐based services and those delivered via one‐to‐one sessions; 3 compared the experiences of those receiving an individually delivered programme with those receiving a group programme.^25^, ^39^, ^46^ Table 2 also illustrates how few of the programmes included exercise sessions^26^, ^37^, ^40^, ^41^ as opposed to just discussion or information about exercise.

**Table 2:** Study, participant and programme details

Study (linked papers)	Participants			Programme		
	No.	% female	Age (years)	Individual or group delivery	Exercise sessions provided	Service model
Ahern et al. (2013)^25^	16	100	Mean 47	Both^a^	×	Commercial/Health service
Allan et al. (2011)^26^	22	75	“Middle aged”	Group	✓	Commercial/Health service/Community
Atkinson et al. (2010)^27^ (27,28)	36	100	25‐39	Individual	×	Health service
Bidgood & Buckroyd (2007)^30^	18	89	n/s	Both^b^	?	Unclear
Bingham et al. (2014)^31^	7	0	47‐63	Individual	×	Community
Brown et al. (2006)^32^	28	64	18 to >75	Both^b^	?	Health service
De Souza & Ciclitira (2005)^33^	8	0	33‐57	Both^b^	?	Commercial
Doyle and Shaw (2012)^34^	11	n/s	n/s	Individual	×	Community
Furness et al. (2011)^35^ (46)	6	100	18‐40	Both^b^	?	Health service
Gray et al. (2013a)^37^ (35,37)	39	0	n/s	Group	✓	Community
Herriot et al. (2008)^39^	46	80	mean 42	Both^a^	c	Commercial
Hunt et al. (2013)^41^	29	0	35‐65	Group	✓	Community
Hunt et al. (2014)^40^	63	0	35‐65	Group	✓	Community
Jones et al. (2007)^42^	24	75	20‐50	Individual	×	Health service
Monaghan (2007)^43^	37	0	16‐79	Group	×	Commercial
Morrison et al. (2014)^44^	20	35	n/s	Individual	×	Community
Penn et al. (2008)^45^	15	47	47‐72	Individual	×	Unclear
Reed et al. (1999)^46^	30	100	Mean 52	Both^a^	c	Health service
Webb et al. (2014)^48^	16	70	n/s	Group	?	Health service
Witty and White (2010)^49^	20	0	n/s	Group	×	Community
Wormald et al. (2006)^50^	16	69	15‐73	Group	×	Community

a Compared views of participants experiencing different programmes, including some which experienced a group programme and some which experienced individual support.

b Participants reflected on a range of previous experiences rather than on a current or specific WMP.

c Comparative study in which some participants received exercise sessions and some did not.

### 3.2 Findings overview

Below we report findings in relation to 2 key questions. First, we report evidence to illustrate *which* components were valued by service users. In the second part of the findings, we address the question as to *how* these programme features are perceived to impact on weight loss outcomes. Whilst, as noted above, WMPs are often described in health promotion guidance or trial reports in relation to education around diet and exercise,^7^, ^9^ these educational aspects were relatively infrequently discussed. Moreover, views were divergent with respect to perceived relevance and utility of such education. Similarly, goal setting, an often cited behaviour change strategy used in WMPs,^7^, ^8^ was mentioned in considerably fewer studies than other components, and positive appraisals of goal setting were also typically less emphatic than for other components. By contrast however, support from providers was the feature discussed most frequently and participants’ views on relationships with providers were consistently positive and highly emphatic; service users were also emphatic about supportive relationships with peers in group services. Service‐user views thus appear to contrast with health guidance and intervention descriptions about critical intervention features, as the notion of support is often secondary or implicit in such descriptions.^35^ Moreover, as the latter finding sections reveal, not only did service‐users value supportive relationships, but they were able to describe *how* such relationships can be critical for changing their behaviour and losing weight.

### 3.3 Which programme components were positively valued by service users?

#### 3.3.1 “It isn't that I need educating, it's more that I need motivating”^25^: dietary advice and goal setting were valued less than supportive relationships

Service users in about half of the studies discussed dietary (n = 12 studies) and exercise (n = 11 studies) features of WMPs, and views on these programme aspects were divergent with respect to perceived relevance and utility. For example, whilst 9 studies included positive service‐user statements about the focus of WMPs on diet,^27^, ^30^, ^31^, ^35^, ^37^, ^39^, ^42^, ^46^, ^48^ participants in 7 studies indicated a perceived lack of need for dietary advice,^25^, ^27^, ^35^, ^37^, ^40^, ^41^, ^42^ for instance:

Perhaps counter to public health assumptions, none of the participants talked about needing an intervention to include education about food, eating, or diet as they believed they already had the necessary knowledge.

25

With regard to physical activity, views were less divergent; nevertheless, participants in 2 studies were clear that they perceived exercise components as of lesser importance.^27^, ^46^ However, these negative opinions may simply reflect that physical activity was less emphasized in the programmes these people had experienced, which may also account for the smaller number of studies in which it was discussed. The notion that the extent of experience of physical activity sessions within WMPs may account for diversity of perceptions about its importance is underscored by one study which compared a range of commercial weight management approaches. This study found that participants on one programme that included a focus on exercise (Rosemary Conley) rated exercise as more important than those receiving other programmes that did not. The authors concluded that “RC [the Rosemary Conley WMP] appeared to have achieved an attitude change towards exercise not observed in the other groups.”^39^ Moreover, within many studies those who reported experience of partaking in physical activity were emphatic about its benefits.^27^, ^30^, ^31^, ^34^, ^37^, ^39^, ^40^, ^44^, ^50^

As noted above, in comparison with other programme features, goal setting was discussed even less frequently (n = 8 studies) than diet and exercise education. Whilst this may simply reflect the types of programmes people were involved with (ie programmes in which goal setting was not emphasized), the lack of emphatic appraisals of goal setting suggests this programme feature is less important to service users. Moreover, even within the small amount of data on goal setting, views were highly divergent. Whilst there seemed to be consensus that goals should be personalized or bespoke,^27^, ^31^, ^35^, ^39^, ^45^ there was no clear pattern with regard to views about *how* goals should be set, or by *whom*. One study described personalized goals being identified and set by providers,^31^ but in 3 studies, service users suggested that they enjoyed setting their own “self‐negotiated” goals.^33^, ^40^, ^44^ In a further study, whilst some participants valued an open or flexible approach, others felt that clear goals prescribed by practitioners would be more helpful: “Some women found the service to be too flexible, and needed more rigid instructions on what and how much to eat.”^27^ A desire to be set strict dietary goals by providers was also voiced by service users in another study.^46^

In contrast, views about provider support were (i) more frequently expressed, (ii) more consistently positive and (iii) more emphatic.

#### 3.3.2 “I need someone to take my hand and take me over”^30^: the importance of provider support

Supportive relationships with WMP providers were the feature of WMPs most frequently discussed by service users (n = 18 studies), and many participants indicated their view that it is a *critical* feature.^25^, ^30^, ^31^, ^32^, ^34^, ^36^, ^39^, ^42^, ^44^, ^50^ Provider relationships were described as an *essential* feature of successful WMPs in 6 studies^25^, ^30^, ^31^, ^34^, ^42^, ^50^; for example, “The most important element of the AL [Active Lifestyles] service appeared to be the AL advisor—the personality and approach of the advisor is likely to determine the success or failure of the service.”^50^ Service users expressed how fervently they felt the need for such support and implied that they were actively seeking it through attendance at WMPs; for example: “I just think I couldn't do it on my own without seeing somebody.”^25^ The emphatic nature of these views, coupled with their extent and unanimity, indicates that provider support is perceived as fundamental to WMP success.

In many studies, and as in the example above, participants linked the value of provider support to the qualities and style of individual providers. Whilst there was a lack of commentary on providers’ professional expertise and experience, participants in many studies emphasized aspects important for relationship building such as providers’ manner or character including

- Friendliness or approachability^25^, ^26^, ^27^, ^31^, ^37^, ^48^, ^50^
- being non‐judgemental^25^, ^27^, ^32^, ^35^, ^45^, ^50^
- empathy and compassion^26^, ^27^, ^44^, ^50^
- being able to communicate verbally^44^, ^50^
- listening,^27^, ^31^, ^50^ and
- being encouraging,^25^, ^31^, ^34^

for example

She says yeah, do you want me to get the scales out or do you want me to leave them in the box. I'm like oh go on, get them out, let's have a look. But I always get the choice and we always have a giggle about it.

26

Service users used phrases like “friendship” and “personal touch” indicating the quality of these relationships, for example

It is more like a friendship relationship with ‘Sarah’ rather than a health person, and you don't feel as though she is instructing you.

50

#### 3.3.3 “I look forward to meeting everyone. We have a laugh”^48^: support from peers

Positive relationships with peers in programmes delivered to groups were also highly valued. For example,

That class motivation I felt worked… building up that…friendly atmosphere and team motivation I found worked quite well

25

Group services were described as attractive for reasons of sociability and fun.^27^, ^31^, ^32^, ^35^, ^37^, ^40^, ^48^ Whilst fewer studies commented on the value of peer support (n = 13 studies) than provider support, this may again be due to types of programmes experienced; as Table 2 illustrates, many studies focused on services provided to individuals rather than groups. However, service users also expressed views that group sessions had negative aspects^25^, ^26^, ^27^, ^30^, ^33^, ^34^, ^37^, ^48^ such as difficulties in raising sensitive issues^27^, ^30^, ^37^ and the embarrassment of group weigh‐ins.^25^, ^27^, ^34^ Thus, there was some lack of consistency in the views presented, but at least some of these views were based on expectations rather than actual experiences of group programmes. Moreover, even though views were mixed, the instances in which group support was positively appraised were informative in understanding how supportive relationships may be crucial for successful weight loss outcomes.

#### 3.3.4 How are the supportive relationships experienced through participation in WMPs perceived to impact on weight loss outcomes?

**Figure 1:**
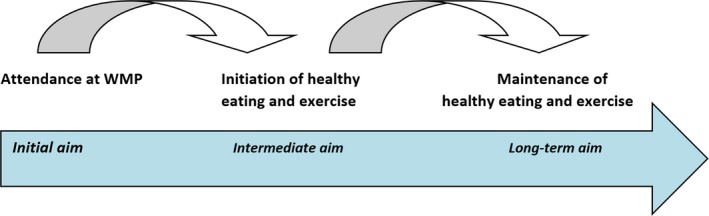
Pathway to self‐regulation [Colour figure can be viewed at http://wileyonlinelibrary.com]

Service users were explicit that supportive relationships with peers and providers helped to motivate attendance at WMPs and initiation of healthy eating and exercise; both of these intermediate outcomes were indicated by service users to be necessary precursors to the ultimate outcome of WMPs, that is successful and sustained weight loss. This pathway to self‐regulation is illustrated Figure 1 below.

### 3.4 Motivations for WMP attendance: social “bonds” with providers and peers

The development of relationships with WMP providers or peers was felt to create the sense of a “bond”; relationships with providers were explicitly linked to programme attendance and retention^26^, ^35^, ^36^, ^44^, ^45^, ^48^ and peer relationships in group‐delivered WMPs were noted for motivating attendance,^31^, ^34^, ^35^, ^36^, ^40^, ^48^ for example,

There was quite a good crack going on all the time, so the following week you kinda felt you wanted to come back and hear how the guys were getting on

36

Many service users had described feeling isolated, depressed or having low self‐esteem or confidence.^27^, ^30^, ^31^, ^32^, ^35^, ^50^ As such, it is perhaps unsurprising that attendance was motivated by the social and psychological benefits of WMPs.^27^, ^30^, ^34^, ^35^, ^40^, ^42^, ^45^, ^50^ Group services were valued for creating a space^31^, ^34^, ^35^, ^37^, ^40^, ^41^, ^49^ with others “in the same boat”^40^; services targeted at specific population groups were particularly valued for creating such spaces; as one participant said of a men's group “The fact that there wasn't going to be any Greek gods in there, it was all going to be human beings, cherubs perhaps, so you're not going to feel out of place.”^37^ Providers were seen as someone to talk to and provide advice,^27^, ^31^, ^32^, ^34^, ^35^, ^42^, ^44^, ^50^ for example “Just the support and knowing that they're there and someone's there to listen to you.”^27^ Providers’ caring attitude was also valued,^25^, ^30^, ^31^, ^32^, ^35^, ^42^, ^44^, ^45^, ^50^ for example “You feel that somebody's batting for you,”^34^ “She used to advise me but so compassionately… she cares so much.”^44^

These findings make clear that for service users, the opportunities for positive social encounters are a powerful draw, a key motivation for turning up for WMP sessions week after week.

### 3.5 Motivation to initiate healthy behaviours: accountability to providers and peers

In addition to enhancing WMP attendance, supportive and caring relationships with providers and peers were felt to foster a sense of accountability.^25^, ^26^, ^27^, ^30^, ^34^, ^35^, ^36^, ^44^, ^45^, ^50^ This feeling of accountability to providers and peers was explicitly described as motivating users to engage in healthy eating and exercise behaviours.

Accountability to providers was seen as a much needed extrinsic motivator, and a catalyst for behaviour change; for example, “For me…what works is the fact that I know…I've got to go and see somebody…and I've got to explain why I haven't lost any weight.”^25^ A positive sense of accountability and its link to engagement in healthy behaviours was also reported in relation to peers in group WMPs^25^, ^35^, ^39^, ^40^ “There was a team spirit and you didnae [did not] want to let the team down.”^40^

Two studies, however, acknowledged that accountability could operate in negative circumstances. In one, service users felt “pressure” to lose weight and employed extreme methods to achieve this, although this specific example occurred in the context of a lack of supportive relationships.^48^ Another study also recognized the potential for accountability to operate in a negative way,^25^ but was explicit that accountability in the context of supportive provider relationships was a much more positive experience.

Crucially, the sense of support and accountability was driven not by the fear of embarrassment that might be associated with peer pressure, but by the feelings of loyalty and obligation

25

Thus, whilst service users felt that these relationships with providers and peers offered social and psychological benefits, they were also clear that such relationships had an impact on health behaviour outcomes.

### 3.6 Motivation for maintenance of healthy behaviours: experience and self‐efficacy

Of course, engaging in health behaviours out of a sense of accountability to others is unlikely to be a long‐term solution. However, as evinced by service‐users’ views, accountability acts as an extrinsic motivator to *begin* to engage in healthy behaviours. This initial engagement allows individuals to experience their own ability to do activities such as exercise, and experience the various benefits afforded by doing it. In turn, these experiences and the development of a sense of self‐efficacy were described as vital for motivating self‐regulation and long‐term maintenance of a healthy lifestyle.

In particular, service users enjoyed partaking in group exercise delivered as part of an intervention, as opposed to just receiving advice. Participants indicated that these experiences increased the chances of engaging in further exercise.^30^, ^37^, ^39^ Service users were also clear that making small initial changes engendered confidence to progress to more active forms of exercise.^27^, ^31^, ^39^, ^41^

“These changes gave some men the ability and confidence to progress to forms of physical activity (such as squash or football) which are more traditionally seen as being valued by men, activities which just weeks before they would have felt unable to contemplate.”

41

In addition to increased self‐efficacy, provision of exercise meant that service users were quite quickly able to appreciate the benefits of exercise. “Because I can see the results you know I've seen my blood pressure go down and I've seen my fitness levels go up.”^39^ In fact, focusing on the fitness gains made through exercise was described both as a psychological boost and as a motivator to further increase exercise in 6 studies,^31^, ^39^, ^41^, ^42^, ^45^, ^50^ for example “It's got me going back to the gym and stuff like that, on top of the walking.”^41^

**Figure 2:**
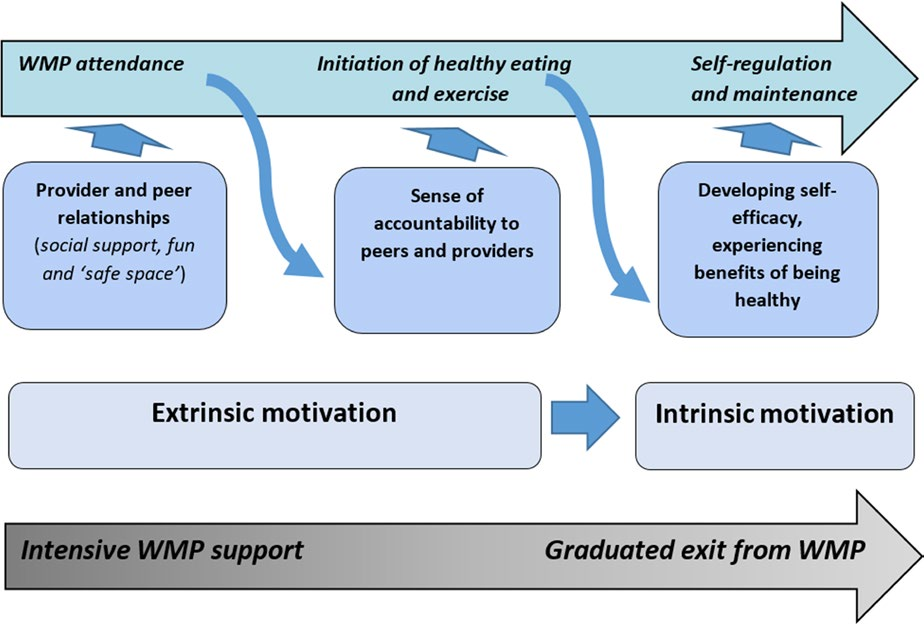
Weight management programmes pathway to successful weight management based on service‐user views [Colour figure can be viewed at http://wileyonlinelibrary.com]

Figure 2 illustrates the various mechanisms that come into play along the pathway from attendance to self‐regulation and the graduated decrease in the level of support needed. The figure illustrates how supportive relationships unlock different mechanisms over time to achieve initial, intermediate and long‐term outcomes. It also illustrates how sources of motivation for achieving initial and intermediate outcomes are extrinsic in nature but that over time these unlock intrinsic motivation for self‐regulation in the longer term. Lastly, the bottom arrow of the figure illustrates how initially intensive support is needed but that overtime, with the unlocking of intrinsic motivation, less support will be needed.

## 4 DISCUSSION

The above findings reveal how at the outset of WMPs, users are seeking a high level of external support. These supportive mechanisms are also implicated in continued programme attendance and the initiation of healthy behaviours. However, it is only once behaviours have been initiated that users are able to perceive the self‐efficacy and enjoy the benefits of behaviour change that lead to self‐regulation. As such, these findings suggest that WMPs will not foster self‐regulation without initially providing a high level of support. As one author concluded:

Participants’ explanatory model appears to suggest weight loss interventions should balance the need to provide a sense of agency while not making the individual entirely responsible for their weight management.

25

It would appear that WMPs which intentionally provide a high level of support at the outset of programmes and build in a graduated exit, with support levels gradually reduced over time, may be an important pathway to effectiveness. In addition, at the initiation stage of a WMP, service users suggested that support and motivation are most important, with educational aspects of the programme (dietary advice, goal setting) only becoming significant at later stages.

This systematic review of the views of service users thus reveals how the development of relationships with providers and peers is an essential first step in a weight management journey. These relationships provide a much needed external motivator or “hook” for people to overcome barriers such as scepticism about the need for dietary advice or a lack confidence to engage in physical activity. As such, the findings suggest existing programme evaluations which focus exclusively or predominantly on the minutiae of programme content, for example, specific diet prescriptions, or on the educational or goal setting aspects of WMPs, may not fully address the needs of service users.

As programmes are seeking to create independent self‐motivated behaviour change and maintenance, it is perhaps understandable that advice and information giving may predominate in programme descriptions.^34^ However, the evidence contained within this review suggests that without the initial extrinsic motivator of supportive relationships, self‐regulation and maintenance are unlikely to be achieved. A review of the management of obesity among men similarly concluded that such “hooks” are necessary and that relationships with providers, peers and others are hugely important to engagement with WMPs.^22^ Moreover, a synthesis of international qualitative research on adults’ weight management experiences also underscores the significance of supportive relationships, concluding that there was “very clearly” a desire for such support.^20^ We do not suggest, of course, that it is unnecessary to incorporate education on healthy diets and physical activity in WMPs, but rather that programme theories need to balance the detail of these educational aspects with a focus on developing supportive and motivational aspects. Indeed, some evidence indicates that less focus on the specifics of dietary advice in programme protocols may be warranted; a comprehensive network meta‐analysis found that most calorie‐reducing diets result in clinically important weight loss *only as long as the diet is maintained*.^51^ Thus, shifting the balance of focus towards understanding what helps people to initiate and maintain positive changes may be a critical step in increasing the chances of success.

In short, the synthesis reveals that WMP features, such as the development of social bonds, not previously considered to be critical components, appear to be fundamental to programme success. It is perhaps not surprising that these less tangible programme features are less emphasized in intervention descriptions. Nevertheless, it appears to be vital that these features are understood and conveyed to those commissioning and delivering programmes if they are to be successful.

As described in the companion article,^19^ when we used QCA to examine the relationships between intervention effectiveness and social support, we discovered that the most effective WMPs were actually characterized by the presence of supportive relationships with providers and peers and that the least effective programmes were characterized by their absence. In addition, WMP features which the views synthesis suggested fostered self‐regulation and maintenance of a healthy weight were also found to be critical for WMP success; increased effectiveness was found to be associated with WMPs offering direct provision of exercise or those initially offering a high level of support but which built in a graduated exit from the programme.

Of course, limitations to the generalizability of our findings are imposed by the very nature of the research included, which is subject to all the biases and confounders that qualitative research is unavoidably prey to. In addition, gaps in the research literature were a further limitation to the generalizability of the review findings. In particular, whilst we did include studies reporting the views of those who had declined to engage in a programme or who had disengaged from the programme, there were very few of these and so the findings predominantly reflect those who successfully engaged with programmes. Similarly, whilst the studies reflected a good range of perspectives in terms of the gender, age and socio‐economic status of participants, we found little or no research from other groups such as minority‐ethnic groups and those with learning disabilities.

The main strength of including qualitative evidence in the overarching review was its ability to reveal critical features of WMPs that were hidden or unanticipated; without the qualitative evidence, such features would have gone untested in the QCA. A qualitative evidence syntheses on obesity management in primary care^17^ reached a similar conclusion, arguing that “approaches to obesity that engage all actors including the public … are needed to coproduce context‐specific solutions to a complex health issue” (p. e246).

The extent to which vital aspects of WMPs, and in particular supportive relationships, are underplayed in published descriptions of interventions was further revealed when conducting the QCA. One of the trials showing greater intervention effects included in the QCA^52^ was accompanied by a process evaluation comprised of qualitative interviews with trial participants^1^ to provide an explanatory account of how weight loss was achieved.^53^ In the (2012) paper,^52^ like many included in the QCA, there was scant detail on supportive relationships reported in the intervention description. The description comprised of a paragraph explaining the programme content relating to physical activity and a paragraph describing the content around nutrition; the notion of provider support or relationships could be inferred only from the very last sentence because of mention of “counselling.” Nevertheless, the qualitative interviews with participants from this trial revealed their view that provider relationships were *the* most critical feature for success. The authors of the study concluded that an “emotional bond” with the service provider provided a “catalytic interaction” that was the *key* to sustainable weight loss.^53^ Because no other trial included in the QCA comprised of an integral process evaluation to uncover the mechanisms behind behaviour change, the findings of this review suggest that further studies of this type are warranted. Other researchers have also noted that an understanding of the determinants of WMP effectiveness is currently inhibited by a failure of researchers to both “describe and investigate the exact content of interventions”^54^ and to conduct process evaluations to investigate the underlying mechanisms of behaviour change.^55^ Indeed, UK‐based trials with integral process evaluations would have been a powerful enhancement of the evidence examined for this review.

Nevertheless, the concordance of the views of participants in a Swedish programme with those of the UK participants involved in the qualitative studies included in this review further underscores the validity of the findings on provider‐user relationships. Moreover, these findings also appear to have resonance with other research findings. For example, a review and meta‐analysis of WMPs conducted in “everyday contexts” found that in contrast to commercial programmes, pooled results from 5 interventions delivered by primary care teams showed no evidence of an effect on weight.^56^ Although not reported in this paper, the views of service providers were also incorporated into our synthesis (see 16 for details); this included the views of primary care‐based providers who described twin pressures of time constraints and inadequate staffing levels, which would inhibit opportunities to develop supportive relationships with patients. Health service and community providers also commonly reported a desire for training specifically to ensure appropriate and sensitive support, further indicating deficiencies in the area of relationship building.^16^ A qualitative synthesis on primary care services for obesity has also called for resource to be allocated specifically to enhancing a sensitive approach to referral and support.^17^

It is recognized that one key value of taking note of patient and public perspectives in research is to “disrupt” and to “complicate our [researchers] assumptions around expertise and knowledge.”^57^ Indeed, evidence has long shown that accessing public and patient perspectives about health services either by involving them directly in the research process^58^ or by including qualitative evidence in systematic reviews^15^, ^59^, ^60^ can enhance service delivery by identifying important but unanticipated or underemphasized intervention mechanisms and components. As such, there have been repeated and urgent calls to better involve patients and the public^57^, ^61^ and for qualitative evidence to enhance systematic reviews of trials.^62^ Whilst this systematic review adds further evidence of how critical such perspectives are, the lack of qualitative process evaluations accompanying the trials included in the QCA underpins claims that patient and public views^57^, ^61^ and qualitative evidence^63^ are still undervalued and underutilized by the medical research establishment.

## 5 CONCLUSION

This systematic review highlights the essential value of patient and public views about the health services they receive, by revealing the mismatch between service‐users’ experiences and perceptions of the critical features of WMPs and the focus of programme descriptions and evaluations. The findings of this synthesis illustrate how supportive relationships with peers and providers are vital to encouraging service‐users’ attendance at WMPs, as well as motivating them to initiate positive behaviour change. The extrinsic motivation of these relationships enabled the behaviour changes necessary for the development of intrinsic motivation, which in turn appears necessary to underpin maintenance and self‐regulation of healthy eating and exercise. The findings thus emphasize the need for research and practitioner communities to balance the current focus on educational components of WMPs with a focus on supportive and motivational aspects. The study thus underscores calls to recognize how vital a holistic understanding of “what happens” in complex psychosocial interventions is and how the perspectives of those with direct experience are essential for improving services as they reveal unanticipated mechanisms or underemphasized intervention features.

## CONFLICT OF INTEREST

None declared.

## Supporting information


** **

## NOTE

1 This study was conducted in Sweden, and so the qualitative component was not eligible to be included in the views synthesis.
