# Supplementary material for: The importance of service‐users’ perspectives: A systematic review of qualitative evidence reveals overlooked critical features of weight management programmes
Source: Health Expect. 2018 Mar 14;21(3):563–73. doi: 10.1111/hex.12657 (PMC5980498; doi:10.1111/hex.12657)
Supplement: Supplementary file 1 [file HEX-21-563-s001.docx]

## Supporting information - Flow of literature through the review: identification of views studies

**Total records**

N = 229

**Duplicate reports removed**

N = 1

N = 2059

**Criteria on which reports excluded (abstract and full text)**

**EX 1 - AGE - not adults (≥18 years)**

**EX 2 - FOCUS - No views, perceptions or beliefs of adults towards weight management programmes for overweight adults**

**EX 3 - COUNTRY - Study not conducted in the UK**

**EX4 - ABSTRACT - conference abstract only**

**EX5 - REVIEW - review of studies**

**EX6 - THIN DATA - views on weight management programmes limited**

**EX 7 - METHODS - Study methods poorly reported**

**Excluded on** **abstract** N= 146

EX 1 - AGE: 6

EX 2 - FOCUS: 46

EX 3 - COUNTRY: 94

**Total records screened**N = 228

**Not obtained on time**

N= 10

**Excluded on full text** N=41

EX 1 - AGE: 0

EX 2 - FOCUS: 17

EX 3 - COUNTRY: 2

EX 4 - ABSTRACT: 6

EX 5 - REVIEW: 4

EX 6 - THIN DATA: 10

EX 7 - METHODS: 2

**Full reports retrieved and screened**

**N = 72**

**Linked reports**

**N = 5**

**Full reports included**

**N = 31**

**Studies included in views synthesis**

N=26

**WMP provider views**

**N = 5**

**Studies of service user views included in synthesis**

N=21
